# Shear Performance and Numerical Simulation of Adhesively Bonded Joints in Multi-Jet Fusion 3D-Printed Polyamide Components

**DOI:** 10.3390/polym17223020

**Published:** 2025-11-13

**Authors:** Frantisek Sedlacek, Martin Stejskal, Nikola Bednarova, Ondrej Spacek

**Affiliations:** Faculty of Mechanical Engineering—Regional Technological Institute, University of West Bohemia, Univerzitni 8, 31200 Pilsen, Czech Republic; stejs55@fst.zcu.cz (M.S.); bednaron@fst.zcu.cz (N.B.); spaceko@fst.zcu.cz (O.S.)

**Keywords:** additive manufacturing, multi-jet fusion, adhesives joints, FEM, polyamide

## Abstract

Additive manufacturing technologies are no longer limited to rapid prototyping but are increasingly used for low-volume production of functional end-use components. Among advanced AM techniques, HP Multi-Jet Fusion (MJF) stands out for its high precision and efficiency. Polyamides, thanks to their balanced mechanical and thermal properties, are commonly used as building materials in this technology. However, these materials are notoriously difficult to bond with conventional adhesives. This study investigates the shear strength of bonded joints made from two frequently used MJF materials—PA12 and glass-bead-filled PA12—using four different industrial adhesives. Experimental procedures were conducted according to ASTM standards. Specimens for single-lap-shear tests were fabricated on an HP MJF 4200 series printer, bonded using a custom jig, and tested on a Zwick-Roell Z250 electro-mechanical testing machine. Surface roughness of the adherends was measured with a 3D optical microscope to assess its influence on bonding performance. The polyurethane-based adhesive (3M Scotch-Weld DP620NS) demonstrated superior performance with maximum shear strengths of 5.0 ± 0.35 MPa for PA12 and 4.4 ± 0.03 MPa for PA12GB, representing 30% and 17% higher strength, respectively, compared to epoxy-based alternatives. The hybrid cyanoacrylate–epoxy adhesive (Loctite HY4090) was the only system showing improved performance with glass-bead-reinforced substrate (16.5% increase from PA12 to PA12GB). Statistical analysis confirmed significant differences between adhesive types (F_3,24_ = 31.37, *p* < 0.001), with adhesive selection accounting for 65.7% of total performance variance. In addition to the experimental work, a finite element-based numerical simulation was performed to analyze the distribution of shear and peel stresses across the adhesive layer using Siemens Simcenter 3D 2406 software with the NX Nastran solver. The numerical results were compared with analytical predictions from the Volkersen and Goland–Reissner models.

## 1. Introduction

Additive manufacturing technology is one of the most rapidly growing areas of industry. Three-dimensional printing technologies represent a revolutionary approach to additive manufacturing, offering a broad range of applications in sectors such as automotive, aerospace, healthcare, and consumer goods. This technology is now very often included in the production of components or their parts for end-users, and not just for non-functional prototypes. This is due to the increasing dimensional accuracy of the printed parts, the quality of their final surface, higher printing speed, larger build size, and better mechanical properties, approaching those of isotropic materials, aa well as increasingly lower production costs.

This means that there has been significant growth in the development and improvement of this production technology in recent years. It also means that many different types of AM methods have been created, each with many different terminologies. The American Society for Testing Materials (ASTM) and the International Organization for Standardization (ISO) have developed several standards of categorization [1]. The standard ASTM/ISO 52900:2015 (Additive Manufacturing—General Principles, Terminology) is one of the primary ones and divides AM technologies into seven main categories of additive fabrication as follows: Material Extrusion, Powder Bed Fusion, Directed Energy Deposition, Vat Photopolymerization, Material Jetting, Binder Jetting, and Sheet Lamination [2].

The increasing deployment of additive manufacturing (AM) technologies for functional components presents designers with novel challenges in dimensioning and utilizing new, highly complex structures and materials. Joints represent one of the most critical aspects of this process, with adhesively bonded joints being particularly advantageous due to growing demands for reduced weight and component size, along with associated emission reductions [3,4]. Adhesive bonding offers an attractive strength-to-weight ratio and is often faster and more cost-effective than conventional mechanical joints [5]. Adhesive joints provide numerous advantages including uniform stress distribution across the bonded area, the ability to join very thin materials, compatibility with similar and dissimilar material types, excellent electrical insulation properties, chemical resistance with minimal electrochemical corrosion between bonded materials, environmental sealing capabilities, and resistance to cyclic loading (fatigue) with vibration-damping properties [6,7]. Despite these advantages, adhesive joints present several limitations, including requirements for specialized fixtures and equipment (presses, ovens, or autoclaves), extensive surface preparation often involving corrosive chemicals, potential esthetic issues (color differences, adhesive excess), service life dependency on environmental conditions (UV exposure, humidity, temperature), and limited upper service temperature thresholds [8,9,10]. Bonding polymer components, particularly those produced through 3D printing, represents a complex process influenced by adhesive type, substrate properties, joint geometry, and, critically, surface preparation. Surface preparation significantly impacts bond strength, with 3D-printed polymer materials often exhibiting surface porosity, heterogeneity, and roughness that can compromise adhesive properties [11,12].

This study is motivated by the growing need for reliable data on the mechanical performance of adhesively bonded joints in parts manufactured by advanced additive manufacturing (AM) systems. In particular, the HP Jet Fusion 4200 series (utilizing Multi-Jet Fusion (MJF) technology) stands among the most advanced industrial 3D printing platforms currently available, offering capabilities that closely approach those of serial production. Due to its ability to consistently produce high-strength, functional polymer components, this technology has become increasingly relevant in both prototyping and end-use applications across various industrial sectors.

Multi-Jet Fusion (MJF) represents a cutting-edge Powder Bed Fusion technology that has emerged as a leading solution for high-precision, functional component manufacturing. Unlike traditional additive manufacturing methods, MJF utilizes the selective application of fusing and detailing agents combined with infrared heating to achieve superior dimensional accuracy and mechanical properties. The technology’s capability to produce isotropic parts with minimal anisotropy makes it particularly suitable for applications requiring reliable mechanical performance across all orientations. Polyamide 12 (PA12) and its glass-bead-reinforced variant (PA12GB) are the primary materials utilized in MJF systems, offering excellent chemical resistance, biocompatibility, and processing characteristics. However, the inherently low surface energy of polyamide materials presents significant challenges for adhesive bonding applications, necessitating systematic investigation of suitable joining methods for complex assemblies and large-scale component manufacturing [13,14,15].

Recent studies have increasingly focused on the adhesive bonding of 3D-printed polymers, with particular attention to MJF technology. Khorasani et al. (2024) provided a comprehensive review of MJF polymer processing, highlighting the unique bonding challenges presented by the layer-by-layer manufacturing process [14]. Wojdat et al. (2025) demonstrated that laser micro-texturing of MJF 3D-printed polymers significantly enhanced adhesion strength in PA12 polyamide adhesive joints, with grid textures providing optimal surface development [16]. Recent research by Ferencik et al. (2024) investigated PA12 surface treatment effects on biocompatibility, revealing that MJF-printed PA12 scaffolds maintain cellular compatibility while showing potential for surface functionalization [15]. Pizzorni et al. (2025) explored adhesive bonding between dissimilar composite materials, including 3D-printed components, providing insights into design solutions for multi-material joints [17]. These contemporary studies confirm the growing industrial relevance of adhesive bonding in additive manufacturing applications and support the need for systematic characterization of MJF-printed polymer joints.

Despite the frequent use of adhesively bonded joints in components fabricated using MJF technology (especially in research laboratories and product development environments), there remains a significant gap in the literature regarding the mechanical behavior of such joints. This is particularly true for shear strength performance and its dependency on the type of adhesive, substrate material, and surface conditions.

The present paper aims to address this gap by providing a comprehensive investigation into the shear strength of bonded joints formed with the following four commercially available structural adhesives: two epoxy-based (Loctite EA 9466 and 3M Scotch-Weld DP490), one hybrid cyanoacrylate–epoxy (Loctite HY 4090), and one polyurethane-based (3M Scotch-Weld DP620NS). These adhesives are applied to two types of polymeric materials processed via HP MJF technology: HP 3D High Reusability PA12 and its composite variant reinforced with glass beads, HP PA12GB.

This work offers a multi-faceted approach to the evaluation of adhesive joint performance. Firstly, the study includes a detailed experimental program that incorporates surface preparation and characterization of adherends, followed by mechanical testing under shear loading to determine adhesive bond strength. Secondly, the experimental results are validated and further examined through advanced numerical simulations based on the finite element method (FEM), allowing for the investigation of stress distributions and potential failure mechanisms within the bonded region. Finally, the study compares the results with predictions from classical analytical models for adhesively bonded joints (specifically the Volkersen model and the Goland–Reissner model) providing insight into the applicability and limitations of these theoretical approaches when applied to modern AM materials and geometries.

Through this comprehensive methodology (combining experimental measurements, numerical simulations, and analytical modeling) the article delivers a robust framework for understanding and predicting the behavior of adhesive joints in polymer components produced by one of the most advanced AM technologies currently available. The findings are expected to contribute to the broader implementation of bonded assemblies in additively manufactured parts intended for structural or semi-structural applications.

In summary the primary objective of this study is to systematically characterize the shear strength performance of adhesively bonded joints in MJF-printed PA12 and PA12GB components using four commercially available structural adhesives. Specific aims include the following:

1. experimental determination of joint shear strength according to ASTM D3163 [18] standard,
2. statistical analysis of adhesive and substrate effects on bond performance,
3. validation of experimental results through finite element analysis incorporating material nonlinearity, and comparison with classical analytical models (Volkersen and Goland–Reissner) to assess their applicability to MJF-printed polymer joints.

The research addresses a critical knowledge gap in the mechanical characterization of adhesive joints for additive manufacturing applications, providing essential design data for industrial implementation. The key achievement is the identification of the best adhesives as optimal joining solutions for MJF-printed polyamide components, with quantified performance differences between neat and glass-bead reinforced substrates that inform material selection strategies for multi-component assemblies.

## 2. Experiment

### 2.1. Adherend Materials

Two types of material were selected for fabrication: HP 3D High Reusability Polyamide 12 (PA12) and glass-bead-filled HP 3D High Reusability Polyamide 12 (PA12GB) composite material. Both materials represent advanced thermoplastic solutions specifically engineered for Multi-Jet Fusion (MJF) technology applications.

PA12 represents one of the most extensively utilized thermoplastic materials in MJF technology, distinguished by its exceptional mechanical properties and processing characteristics. This semi-crystalline engineering thermoplastic demonstrates a remarkable tensile strength of approximately 48 MPa, a tensile modulus of 1650–1800 MPa, and an elongation at break of 15–20%, depending on build orientation [19,20]. The material exhibits excellent chemical resistance to oils, greases, aliphatic hydrocarbons, and alkaline solutions, making it particularly suitable for applications exposed to demanding environmental conditions [21]. The fine powder granulometry, with a median particle size of 60 μm, enables the production of components with precise dimensional accuracy and superior surface finishes. PA12 demonstrates outstanding dimensional stability due to its low moisture absorption characteristics, with water absorption rates of approximately 0.8% under normal climatic conditions. The material’s melting point of 187 °C and relative high heat deflection temperature of 175 °C at 0.45 MPa provide adequate thermal performance for most engineering applications. Research indicates that PA12 exhibits excellent biocompatibility (meeting USP Class I–VI requirements) and US FDA guidance for intact skin surface devices. The material density of 1010 kg/m^3^ contributes to lightweight component design while maintaining structural integrity. Additionally, PA12 demonstrates superior powder reusability characteristics, with up to 80% surplus powder reuse capability while maintaining consistent mechanical properties across multiple build cycles [19].

PA12GB represents a reinforced composite formulation incorporating 40% glass beads as a strengthening filler material. This additive significantly enhances the material’s mechanical properties, particularly stiffness and dimensional stability characteristics. Research demonstrates that glass-bead incorporation increases tensile modulus by 85% and flexural modulus by 36% compared to unfilled PA12, while reducing tensile strength by 39% and flexural strength by 14%. The composite material exhibits superior dimensional stability through reduced moisture absorption and increased rigidity, enabling components to maintain their geometric integrity under mechanical or thermal stress conditions. Studies indicate that glass-bead-filled PA12 composites demonstrate excellent long-term wear resistance and enhanced thermal performance compared to unfilled PA12 [19,22]. The percentage crystallinity of the glass-bead PA12 composite is approximately 24%, compared to 31% for PA12-only parts. This reduction in crystallinity is attributed to the incorporation of glass beads inducing heterogeneous nucleation, which affects the polymer crystallization behavior. This type of build material maintains excellent powder reusability characteristics too (achieving up to 70% surplus powder reusability while delivering consistent performance).

Both HP 3D High Reusability PA12 and PA12GB represent advanced material formulations specifically engineered for Multi-Jet Fusion additive manufacturing. PA12 offers exceptional versatility, toughness, and surface finish quality, making it suitable for detailed functional components and prototyping applications. PA12GB provides enhanced stiffness and dimensional stability, positioning it as the preferred choice for load-bearing components and applications subjected to elevated mechanical stress conditions. The material selection process should comprehensively evaluate application-specific requirements, including mechanical loading conditions, environmental exposure, and functional performance criteria to ensure optimal component performance and reliability.

### 2.2. Adherend Fabrication

The single-shear lap specimens were fabricated using the HP Jet Fusion 4200 series 3D printer, which utilizes Multi-Jet Fusion (MJF) technology developed by Hewlett–Packard and commercially introduced in 2016. This additive manufacturing process employs powdered material that is conveyed from a reservoir by an Archimedean screw and uniformly distributed onto the build platform via a rotating roller. Subsequently, according to the programmed geometry, print heads equipped with precision nozzles deposit functional agents onto specific regions of the powder bed, analogous to inkjet printing. Two types of agents are employed: a fusing agent, which is selectively applied to areas where the material is intended to coalesce, and a detailing agent, which is deposited along the periphery of the part geometry to facilitate thermal insulation, improve dimensional accuracy, and enhance surface finish. Upon application, the powder bed is exposed to infrared (IR) radiation. The regions coated with the black fusing agent—due to their higher IR absorption—undergo localized melting and solidification, forming the initial layer of the part. In contrast, uncoated areas remain unaffected. The build platform is then incrementally lowered, and the next powder layer is applied using the same deposition mechanism. This layer-by-layer process continues iteratively, based on the input 3D model, until the complete part geometry is achieved. Post-processing involves vacuum removal of residual unfused powder, which is collected and recycled for subsequent builds. The system is capable of printing layers with a nominal thickness of 80 μm in approximately 10 s per layer. The maximum build volume is 380 × 284 × 380 mm^3^, allowing a full build cycle to be completed in approximately 11.5 h. The basic properties of PA12 and PA12 with glass bead powder are given in Table 1.

### 2.3. Surface Preparation

Bonding of polymer components, including those fabricated via additive manufacturing, constitutes a multi-faceted process governed by adhesive chemistry, substrate characteristics, joint configuration, and, critically, surface conditioning. Among these factors, surface preparation exerts a pivotal influence on bond integrity. Three-dimensional-printed polymers typically present inherent porosity, heterogeneity, and micro-roughness, all of which can impair adhesive wetting and interfacial adhesion [24]. Mechanical abrasion enhances interfacial contact by enlarging the effective bonding area and promoting mechanical interlocking between the adhesive and substrate [25]. However, excessive roughness may introduce stress concentrators within the microstructure, ultimately diminishing bond strength. Therefore, optimal roughness parameters must be empirically determined for each adherend (polymer)–adhesive pair. Chemical primers further improve adhesion by increasing surface free energy and facilitating covalent or secondary-force interactions at the interface [24]. Similarly, non-thermal plasma or corona treatments activate polymer surfaces by introducing polar functional groups and elevating surface energy, thereby enhancing wettability and adhesive bonding without altering bulk properties [26]. In practice, a combined surface treatment strategy—for example, light sanding followed by plasma activation yields synergistic improvements in bond strength, as mechanical interlocking and chemical activation act in concert to maximize interfacial adhesion. Consequently, tailoring surface preparation protocols to the specific polymer substrate and adhesive system is essential for achieving robust, durable joints in both conventional and 3D-printed polymer assemblies [24].

Recent research emphasizes the critical influence of surface chemistry and interfacial design in additively manufactured polymer joints. Chen et al. (2023) demonstrated that plasma activation significantly enhanced bond strength in MJF-printed PA12 parts [27]. In addition, fatigue studies on polymeric single-lap joints (e.g., Musiari & Moroni, 2021) show that surface pretreatments influence both static and fatigue performance [28]. These findings align with our aim to systematically investigate adhesive bonding in AM polymer substrates.

In this case, the specimens were treated using a Normfinish AM TUMBLER DL by the Normfinish company located in Hengelo, The Netherlands and equipped with an automatic rotary drum. The blasting process employed MF 30/40 abrasive, applied at an air pressure of 3.5 bar. This abrasive is composed of thermoplastic acrylic and plastic blends of the melamine–formaldehyde type and is specifically designed for the surface treatment of plastic components without causing structural loss or damage. The technical specifications of the abrasive are provided in Table 2.

**Figure 1:**
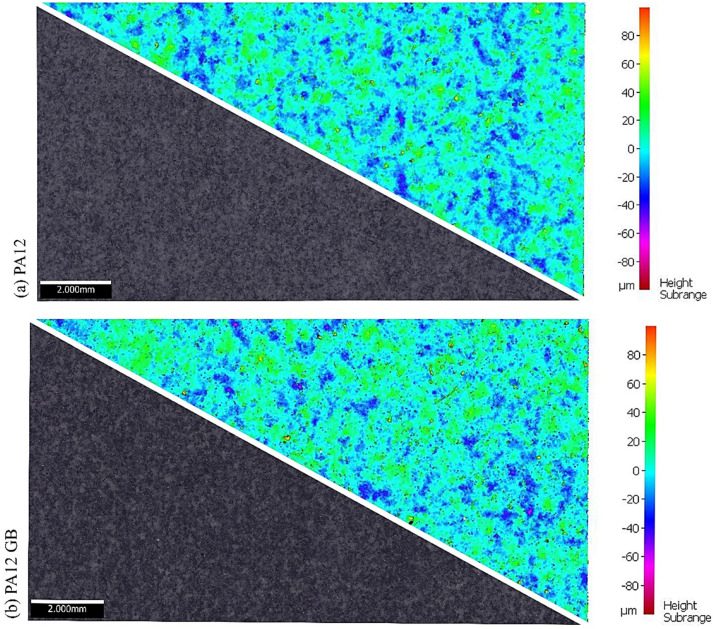
Surface texture and height subrange of vertically printed specimens: (**a**) HP MJF PA12 and (**b**) HP MJF PA12 GB.

The surface quality of additively manufactured parts is a critical factor influencing their mechanical performance, bonding behavior, and functional integration. Numerous studies confirm that print parameters and reinforcement loading substantially affect surface roughness in MJF-printed thermoplastics. The surfaces of specimens printed vertically from material PA12 and PA12 GB were measured. Durface topography was measured non-contact using the Alicona IFM G4, a high-precision optical profilometer capable of capturing three-dimensional surface features with submicron vertical resolution. The device enabled measurement of key roughness parameters (such as Ra, Rz, and Rq) and facilitated distinct mapping of surface texture over representative areas. Surface textures and the height subrange of the specimens are given in Figure 1.

**Figure 2:**
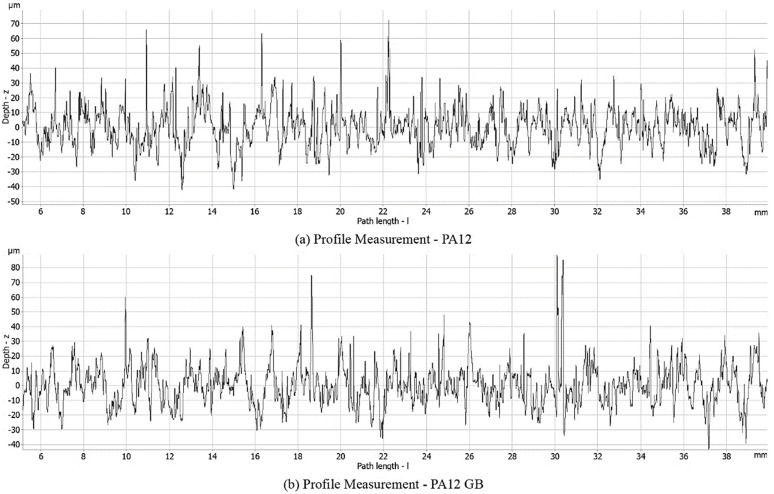
Charts of profile measurement for (**a**) HP MJF PA12 and (**b**) HP MJF PA12 GB.

The surface roughness was determined from profile measurement for both materials. The average profile roughness of 10.69 μm was found for PA12. An average profile roughness of 10.19 μm was measured for PA12 with glass beads, which is 4.5% lower. The characteristics of the profile measurement of the materials are given in Figure 2. Other parameters are given in Table 3.

The relationship between surface roughness and bonding performance in MJF-printed substrates is complex and substrate-dependent. The measured surface roughness values (Ra = 10.69 μm for PA12, Ra = 10.19 μm for PA12GB) fall within the optimal range for mechanical interlocking with structural adhesives, which typically perform best on surfaces with Ra values between 5 and 25 μm. The 4.5% lower roughness of PA12GB compared to neat PA12 correlates with the mixed adhesive performance observed, where some adhesives (DP620NS) performed better on the smoother PA12 surface, while others (HY4090) showed superior bonding to the slightly rougher PA12GB substrate. This substrate-dependent behavior suggests that the glass-bead reinforcement affects not only surface topography but also local surface chemistry and energy characteristics. The relatively high surface roughness of both substrates promotes mechanical interlocking, contributing to the consistent adhesive failure mode observed across all specimens. However, the lack of cohesive failure indicates that chemical bonding remains limited despite adequate mechanical interlocking, highlighting the need for surface activation treatments to achieve optimal bond strength in polyamide substrates.

### 2.4. The Adhesives Tested

In the context of bonding polyamide (PA12) substrates, solvent-based adhesives are generally acknowledged as the most effective option. Nevertheless, a variety of commercial structural adhesives are also frequently employed, including epoxy resins (and their hybrids), phenolics (such as nylon–phenolic and nitrile–phenolic variants), cyanoacrylates, nitriles, neoprene, and polyurethane formulations [29].

For this study, the following four structural adhesives were selected for bonding both PA12 and PA12 GB substrates:

- two epoxy-based adhesives: Loctite EA 9466 and 3M Scotch-Weld DP490,
- one hybrid cyanoacrylate–epoxy adhesive: Loctite HY 4090, and
- one polyurethane adhesive: 3M Scotch-Weld DP620NS.

**Epoxy-Based Adhesives**

Epoxies—commercially introduced in the mid-20th century—remain one of the most widely used classes of structural adhesives due to their excellent tensile and shear strength, low shrinkage, high resistance to moisture and oils, and strong adhesion to diverse substrates. However, they generally exhibit limited peel strength in their unmodified (clear) form and often require post-curing to attain their full mechanical potential [30].

- Loctite EA 9466 represents a high-toughness, two-component epoxy adhesive system featuring room temperature curing and exceptional shear strength with enhanced peel resistance. This industrial-grade adhesive system demonstrates superior chemical resistance across a broad spectrum of chemical environments and functions as an excellent electrical insulator. The adhesive exhibits a 2:1 volume mixing ratio with a 60 min working life and 180 min fixture time, making it particularly suitable for applications requiring extended assembly times. The technical specifications indicate excellent bond strength performance on diverse plastic and metal substrates, with a peel strength of 8.0 N/mm on grit-blasted steel [31]. The medium-viscosity formulation (15,000–50,000 mPa·s for the resin component) facilitates controlled application while maintaining gap-filling capabilities.

**Modified Epoxy**

Epoxy formulations are readily tailored through the incorporation of elastomers, reactive agents, and fillers to enhance toughness, peel strength, cohesion, and thixotropy.

- 3M Scotch-Weld DP490 constitutes a black, thixotropic, two-component epoxy adhesive engineered for high-strength structural applications. This formulation demonstrates exceptional temperature resistance up to 120 °C, coupled with outstanding shear and peel strength characteristics and superior impact durability. The adhesive’s 2:1 volume mixing ratio and 90 min working life provide adequate processing time for complex assembly operations [32]. The thixotropic properties enable vertical surface applications without sagging, while maintaining excellent bonding performance on challenging substrates including powder-coated metals and various thermoplastics. The adhesive demonstrates superior environmental resistance and stability under both static and dynamic loading conditions.

**Polyurethane Adhesives**

Two-component polyurethane adhesives yield lightly cross-linked thermoset bonds characterized by flexibility along with considerable peel and shear strength, making them well-suited for plastic bonding where peel resistance is crucial [33].

- 3M Scotch-Weld DP620NS is a flexible structural polyurethane adhesive noted for excellent impact resistance, low-temperature performance, low shrinkage, low viscosity, and effective adhesion to a wide range of substrates including plastics, composites, metals, ceramics, glass, and rubber [34]. The 1:1 mixing ratio and 20 min working life facilitate straightforward application procedures, while the non-sag paste formulation enables precise handling during assembly operations. The adhesive demonstrates effective bonding to diverse substrates including composites, metals, wood, plastics, rubbers, ceramics, and glass, though primer application may be required for optimal durability on certain substrates.

**Cyanoacrylate Adhesives**

Cyanoacrylates—commonly known as “superglues”—form thermoplastic bonds quickly and with minimal thickness, providing high bond strength and rapid set time. However, they are typically brittle, exhibiting low impact and peel resistance and poor resistance to heat and moisture [35]. These drawbacks can be mitigated through hybrid formulations.

- Loctite HY 4090 is a cyanoacrylate–epoxy hybrid adhesive that maintains high temperature resistance (up to 150 °C), chemical and moisture resistance, and robustness against shock and vibration. Its thixotropic nature enables effective bond filling in joints up to 5 mm thick, accommodating rough or imperfect adherend surfaces; its primary properties are listed in Table 4.

The key advantages of each selected adhesive are listed in Table 4.

This selection aligns with the current consensus in structural adhesive technology, which recognizes that epoxy systems offer the broadest adaptability and performance for bonding PA12, while polyurethane and cyanoacrylate–epoxy hybrids provide flexibility and rapid curing, respectively. The main physical properties of the adhesive are given in Table 5 [36].

### 2.5. Test Methods

ASTM D3163 (Standard Test Method for Determining Strength of Adhesively Bonded Rigid Plastic Lap-Shear Joints in Shear by Tension Loading) [18], which is derived from ASTM D1002 (Standard Test Method for Apparent Shear Strength of Single-Lap-Joint Adhesively Bonded Metal Specimens by Tension Loading) [38], was used to define the specimen geometry and conduct experimental testing. Unlike D1002, D3163 permits the use of substantially thicker adherends, a critical adaptation given that P12 and PA12 GB substrates would otherwise fail in the adherend material before the adhesive joint reached its maximum load. Consequently, ASTM D3163 [18] ensures that measured failures occur within the bonded interface rather than in the plastic adherend itself.

**Figure 3:**
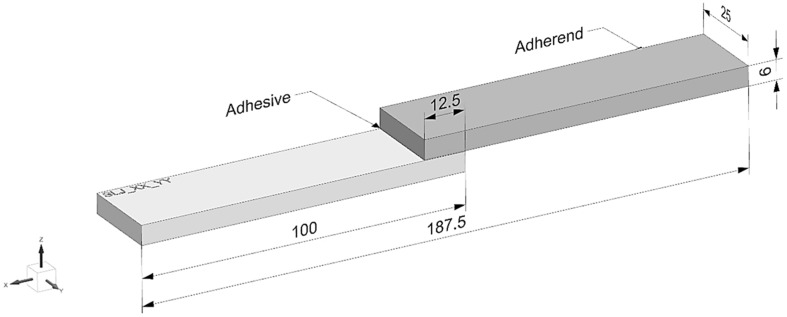
Geometrical parameters of single lap-shear specimen for experimental testing.

The single-lap shear specimens were fabricated with adherend arms measuring 100 mm × 25 mm × 6 mm and an overlap length of 12.5 mm, as shown in Figure 3.

The specimen adherends were produced directly via Multi-Jet Fusion (MJF) 3D printing (see Section 2.2). Prior to mechanical testing, all specimens were conditioned at ambient laboratory conditions for one week to ensure full development of adhesive strength, in accordance with manufacturer recommendations. Bonding was performed using a custom-designed alignment jig that precisely controls adherend parallelism, overlap length, and bondline thickness by permitting fine adjustment of the upper adherend position.

For each adhesive–substrate combination (Loctite EA 9466, 3M Scotch-Weld DP 490, Loctite HY 4090, and 3M Scotch-Weld DP 620NS bonded to both PA12 and PA12 GB), four lap-shear specimens were prepared, yielding 32 specimens in total from 64 printed adherends. The average adhesive bondline thickness ranged from 0.189 mm to 0.228 mm and overlap length l ranged from 12.1 to 12.4 mm, overlap width b ranged from 24.8 to 25.1 mm. Prior to bonding, adherend surfaces were degreased using Loctite SF 7063 industrial cleaner. After curing, excess adhesive was carefully removed, with particular attention to the specimen edges to eliminate unintended filets (parasitic connections).

**Figure 4:**
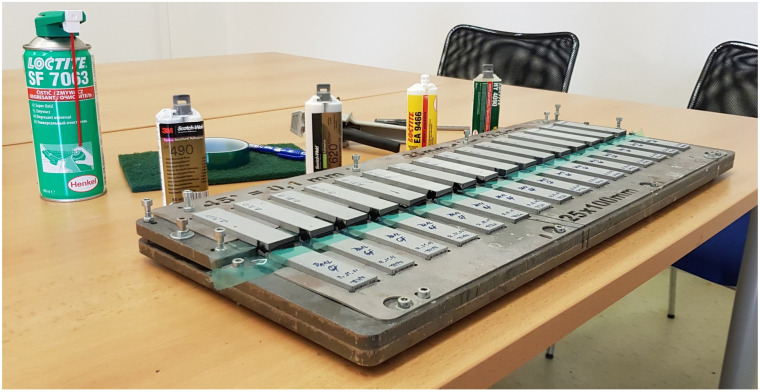
The special jig for accurately gluing the specimens.

The specimen preparation methodology employed in this study follows established ASTM D3163 [18] protocols specifically designed for adhesively bonded plastic lap-shear specimens. The approach of bonding individual pre-cut adherends rather than large panels followed by sectioning was deliberately chosen to ensure consistent bondline thickness control and minimize residual stresses that can arise from post-bonding machining operations. Large-panel bonding followed by waterjet or mechanical cutting, while potentially providing more uniform bondline thickness, introduces several complications for polymer substrates: (1) potential thermal damage from cutting operations, (2) edge effects from cutting fluid interaction with the bondline, and (3) difficulty in maintaining specimen alignment during sectioning. The custom alignment jig employed in this study (Figure 4) provided precise control over bondline thickness (CV < 8%) and overlap geometry, ensuring statistical validity across specimen populations. The methodology is consistent with industry practice for polymer adhesive testing and enables direct comparison with the extensive literature data. Post-bonding machining of polymer joints often introduces micro-damage at specimen edges that can act as crack initiation sites, potentially compromising the validity of strength measurements. The direct bonding approach eliminates these concerns while maintaining the statistical rigor required for comparative adhesive evaluation.

The adherends were glued without the use of applied pressure. The decision to perform bonding without applied pressure was based on manufacturer specifications for the selected adhesives and the requirements of the ASTM D3163 [18] standard testing. The structural adhesives used in this study (epoxy-based DP490 and EA9466, polyurethane DP620NS, and hybrid HY4090) are formulated as contact-pressure systems that develop full strength through chemical curing rather than pressure-induced consolidation. Applied pressure during curing can actually be detrimental for these systems, as follows: (1) expelling adhesive from the joint and creating starved bondlines, (2) introducing non-uniform stress states that affect curing kinetics, and (3) creating residual stresses that can compromise joint performance. The thixotropic nature of DP490 and HY4090 specifically prevents sagging and enables gap-filling capabilities that would be compromised under external pressure. The controlled bondline thickness achieved through the alignment jig (0.19–0.23 mm range) demonstrates adequate adhesive retention without pressure application. This methodology aligns with industrial adhesive bonding practices for these adhesive types and ensures that the measured strengths represent realistic performance under practical application conditions. The absence of applied pressure also eliminates variables related to pressure magnitude and distribution that could affect inter-laboratory reproducibility of results.

Adhesive squeeze-out and filet formation at overlap edges were carefully controlled during specimen preparation to minimize their influence on joint strength measurements. Excess adhesive was systematically removed after the initial cure using precision tools, with particular attention to maintaining consistent edge conditions across all specimens. However, complete elimination of edge filets is practically impossible, and some residual adhesive remains at specimen edges, which can contribute to apparent joint strength through stress redistribution mechanisms. Previous research indicates that adhesive filets can increase apparent joint strength by 10–20%, depending on filet geometry and adhesive properties [39]. The controlled filet removal process ensures that comparative rankings between adhesives remain valid, as all specimens experienced similar edge condition processing. While absolute strength values may include filet contributions, the systematic approach employed enables reliable comparative evaluation of adhesive performance. Future studies incorporating precise filet geometry control or numerical modeling of filet effects would enhance the accuracy of strength predictions for industrial applications where filet conditions may vary.

**Figure 5:**
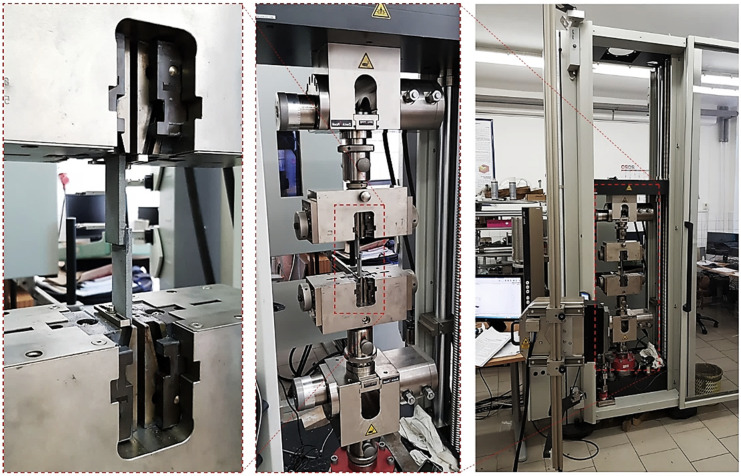
Experimental measurement of shear strength of specimen—Zwick-Roell Z250 tensile machine (**right**); detail of self-locking jaw (**center**); detail of attachment of the specimen (**left**).

The shear strength of the lap-shear specimens was determined using a Zwick-Roell Z250 electro-mechanical tensile testing machine by ZwickRoell GmbH & Co. KG located in Ulm, Germany and equipped with a 5 kN load cell. The instrument undergoes routine calibration in accordance with EN ISO 9513:2002 (Calibration of Extensometer Systems) and EN ISO 7500-1:2004 (Verification of Uniaxial Testing Machines) to ensure measurement accuracy and traceability. Specimens were gripped in serrated mechanical jaws (each 25.4 mm in length) with a deliberate offset arrangement to eliminate bending moments arising from clamping. All tests were conducted at a constant crosshead speed of 0.05 in/min (1.27 mm/min), as specified by ASTM D3163 [18] for polymer lap-shear testing. Ambient laboratory conditions were maintained at 21 °C and 42% relative humidity throughout testing. The Zwick-Roell testXpert II 2024 software suite controlled the test sequence and performed data acquisition and post-processing. Figure 5 illustrates the complete testing apparatus, including a detailed view of specimen alignment and grip configuration during shear loading.

In this case, the resulting reaction force (or subsequently calculated stress in lap shear) was evaluated against the displacement of the crosshead. The experimental measurement of displacement represents crosshead movement rather than localized strain distribution within the overlap region, which is acknowledged as a limitation of the current study. Direct measurement of deformation along the overlap length would require advanced techniques such as digital image correlation (DIC) or strain gauge arrays positioned at multiple locations across the joint. The crosshead displacement measurements provide valuable information for comparative analysis of joint stiffness and ultimate strength, enabling ranking of adhesive performance and validation of failure loads predicted by analytical and numerical models. However, for detailed understanding of strain distribution and progressive failure mechanisms, localized measurement techniques would be required. Future investigations should incorporate DIC analysis to capture the evolution of strain fields during loading, particularly in the critical overlap edge regions where stress concentrations initiate failure. The current approach aligns with standard ASTM D3163 [18] testing protocols and provides data suitable for engineering design applications, while acknowledging that more sophisticated measurement techniques would enhance the fundamental understanding of joint behavior and failure progression mechanisms.

### 2.6. Results of Experimental Testing

The shear strength (*τ*) of each lap-shear specimen was determined by dividing the maximum load at failure (*F_max_*) by the adhesive bond area (*A_bond_*), as expressed by the equation as follows:

(1) $$\tau =\frac{F_{max}}{A_{bond}}\;\left[MPa\right]$$

Reported values represent the shear strength results of all specimens that failed in accordance with the relevant ASTM failure mode criteria (i.e., cohesive or adhesive failure within the bonded interface).

Scanning electron microscopy (SEM) analysis was conducted on representative fracture surfaces using a TESCAN VEGA 3 microscope by TESCAN Group a.s. located in Brno, Czech Republic, to confirm failure mechanisms and validate the exclusively adhesive failure mode observed in all specimens. The SEM micrographs revealed clean separation at the adhesive–substrate interface with no residual adhesive remaining on the PA12 or PA12GB surfaces, confirming pure interfacial failure rather than cohesive or mixed-mode failure. The fracture surfaces exhibited characteristic features of adhesive debonding, including smooth substrate surfaces with minimal surface damage and absence of adhesive residues. This failure mode indicates insufficient chemical bonding between the adhesives and the MJF-printed substrates, likely due to the inherent low surface energy of polyamide materials and the presence of residual processing agents from the MJF printing process. The consistent adhesive failure across all adhesive types suggests that surface preparation methods more aggressive than plastic media blasting may be required to achieve cohesive failure and maximize joint strength potential.

**Figure 6:**
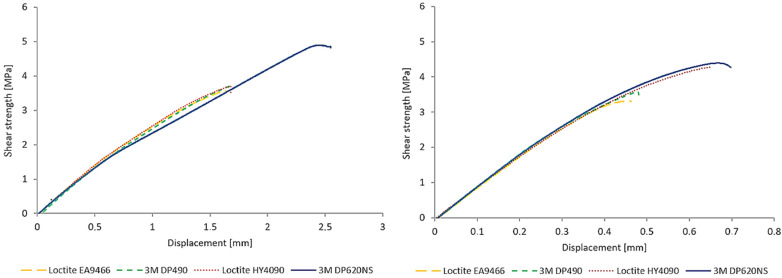
Average shear strength–displacement curves of selected adhesives—PA12 adherend (**left**) and PA12 GB adherend (**right**).

Figure 6 presents representative shear strength–displacement curves for each adhesive–substrate combination, illustrating both ultimate strength and displacement at failure.

This section reports tensile shear strength results for four structural adhesives bonded to PA12 and PA12GB adherends, analyzed using two-way ANOVA with interaction to assess the main effects of adhesive and substrate and their interaction on the response y (MPa) in a balanced 4 × 2 factorial design with four replicates per cell (n=32). The model is as follows:

(2) $$y_{ijk}=\mu +\alpha_{i}+\beta_{j}+(\alpha \beta {)}_{ij}+\varepsilon_{ijk},$$

where μ is the grand mean, $\alpha_{i}$ represents the fixed effect of the *i*-th adhesive level, $\beta_{j}$ represents the fixed effect of the *j*-th adherent level, $(\alpha \beta {)}_{ij}$ is the interaction effect between adhesive and adherent, and $\varepsilon_{ijk}∼N(0,\sigma^{2})$ is the random error term assuming normality, homoscedasticity, and independence. Subscripts indicate i ∈ {1, 2, 3, 4} for adhesives {DP620, DP490, EA9466, HY 4090}, j ∈ {1, 2} for adherents {PA12, PA12GB}, and k ∈ {1, 2, 3, 4} for replicates within each cell.

Cell means (MPa) show clear ranking differences across adhesives and substrates; means ${\overset{ˉ}{y}}_{ij}$ are computed from four replicates per cell and correspond to specimen sets. Standard deviations $s_{ij}$ within cells range from 0.135 to 0.245 MPa, indicating acceptable measurement precision. Mean tensile shear strength and standard deviations are given in Table 6.

Marginal means across adherents are as follows: 3M Scotch-Weld DP620NS (4.698 MPa), Loctite HY 4090 (4.167 MPa), 3M Scotch-Weld DP490 (3.669 MPa), and Loctite EA 9466 (3.653 MPa). Marginal means across adhesives are as follows: PA12 (4.055 MPa) and PA12GB (4.039 MPa), with a grand mean of ${\overset{ˉ}{y}}_{..}=4.047$ MPa.

**ANOVA Statistical Parameters and Outcomes**

The analysis partitioned total variation SST=8.958 into components attributable to main effects and interaction. The sum of squares (SS) represents variability, degrees of freedom (df) indicate independent information units, mean squares (MS = SS/df) provide variance estimates, F-ratios test effect significance against error variance, and *p*-values quantify statistical evidence.

Degrees of freedom partition are as follows: $df_{A}=a-1=3$ for the adhesive factor (four levels), $df_{B}=b-1=1$ for the adherent factor (two levels), $df_{AB}=(a-1)(b-1)=3$ for interaction, and $df_{E}=ab(n-1)=24$ for error (4 replicates × 8 cells − 8), totaling $df_{T}=abn-1=31$. The error mean square ${\mathrm{MS}}_{E}=0.063$ MPa^2^ serves as the denominator for all F-tests, representing within-cell variance ${\hat{\sigma }}^{2}$. Results of the two-way ANOVA are given in the Table 7.

Effect sizes (η^2^ = SS_effect/SS_total) indicate that adhesive type accounts for 65.7% of total variance, interaction explains 17.5%, while adherent type contributes negligibly (0.02%). The highly significant adhesive effect $F_{3,24}=31.37,\;p<0.0001$ demonstrates substantial differences among adhesive means beyond random variation. The significant interaction $F_{3,24}=8.35,\;p<0.001$ indicates that adhesive ranking depends on substrate, violating the assumption of consistent main effects.

**Comparative Adhesive Performance**

The highest shear strength was observed for polyurethane adhesive 3M Scotch-Weld DP620, with average values of 5.00 MPa on PA12 and 4.39 MPa on PA12GB. Compared to the next-strongest adhesive, hybrid cyanoacrylate–epoxy Loctite HY 4090 (3.85 MPa on PA12 and 4.49 MPa on PA12GB), DP620 exhibited a 30.0% higher shear strength on PA12 and a 2.2% lower strength on PA12GB.

Loctite HY 4090 ranked second, showing 3.85 MPa on PA12 and 4.49 MPa on PA12GB—an increase of 16.5% when bonding PA12GB vs. PA12.

**Figure 7:**
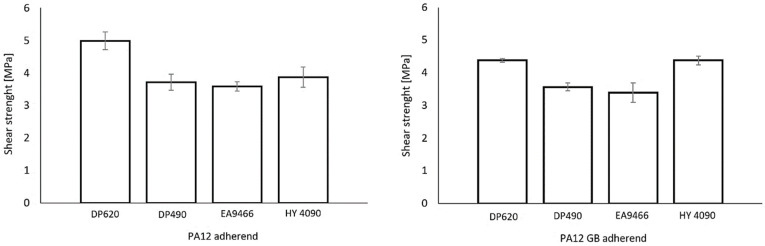
Comparison of average shear strength—PA12 adherend (**left**) and PA12 GB adherend (**right**).

The two epoxy adhesives exhibited the lowest strengths and were nearly identical across substrates. 3M Scotch-Weld DP490 achieved 3.71 MPa on PA12 and 3.63 MPa on PA12GB, which corresponds to a 25.8% decrease relative to DP620 on PA12 and a 17.4% decrease on PA12GB. Loctite EA 9466 recorded the lowest values, 3.66 MPa on PA12 and 3.65 MPa on PA12GB, representing decreases of 26.9% and 16.9% relative to DP620, respectively. The substrate effect (PA12GB vs. PA12) ranged from +16.5% for HY 4090 (i.e., stronger on PA12GB) to −12.2% for DP620 (i.e., stronger on PA12). Figure 7 compares the average shear strengths for all adhesive–substrate combinations.

**Variables and Measurement Specifications**

- Response variable $y_{ijk}$: tensile shear strength in MPa (equivalent to N/mm^2^) calculated as maximum force divided by bonded area according to ASTM D1002 [38] methodology.
- Factor A (Adhesive): four levels representing structural adhesive types—DP620 (polyurethane), DP490 (epoxy), EA9466 (epoxy), and HY 4090 (hybrid cyanoacrylate–epoxy)—treated as fixed effects.
- Factor B (Adherent): two levels representing substrate materials—PA12 (neat polyamide 12) and PA12GB (glass-bead-filled polyamide 12)—treated as fixed effects.
- Replication parameter k: four independent specimens per factor combination with systematic identification codes LS_XX_YY (PA12) and SL_XX_YY (PA12GB), where XX denotes adhesive group and YY denotes replicate number.
- Geometric parameters: overlap length L (12.1–12.4 mm), overlap width b (24.8–25.1 mm), bonded area A=L×b (300.08–311.24 mm^2^), and adhesive thickness $t_{gluing}$ (0.189 mm to 0.228 mm).
- Error variance $\sigma^{2}$: estimated by ${\mathrm{MS}}_{E}=0.063$ MPa^2^, representing within-cell measurement variability and experimental uncertainty.
- Number of samples: the sample size of four specimens per adhesive–substrate combination (*n* = 32 total) was determined based on standard practice in adhesive testing and preliminary power analysis for detecting meaningful differences in joint strength. ASTM D3163 [18] recommends a minimum of 3–5 specimens per test condition, with four specimens providing a reasonable balance between statistical power and experimental efficiency. Power analysis indicated that with the observed standard deviations (0.13–0.30 MPa) and four replicates, the study achieves sufficient statistical power (β > 0.80) to detect differences of 0.5 MPa between adhesive means, which represents a practically significant performance difference (>10% relative change). The coefficient of variation for most specimen groups remained below 10%, indicating acceptable measurement precision for engineering applications. Bootstrap resampling analysis of the data confirmed that the confidence intervals calculated from four specimens provide reliable estimates of population means, with minimal bias compared to larger sample sizes. While larger sample sizes would enhance statistical precision, the four-specimen approach enables comprehensive factorial investigation within practical resource constraints while maintaining statistical validity. The consistent trends observed across multiple adhesive–substrate combinations support the reliability of the conclusions drawn from this sample size.

Statistical analysis of substrate effects reveals significant variations depending on adhesive type, as confirmed by the significant adhesive–substrate interaction (F_3,24_ = 8.35, *p* = 0.001). For the DP620NS adhesive, PA12 demonstrated 12.2% higher strength than PA12GB (5.00 vs. 4.39 MPa), with the difference being statistically significant (*p* = 0.003) based on Tukey’s post hoc analysis. This contradicts the initial observation and emphasizes the importance of proper statistical evaluation. Conversely, HY4090 showed significantly higher performance with PA12GB (4.49 MPa) compared to PA12 (3.85 MPa), representing a 16.5% increase (*p* = 0.002). The epoxy adhesives (DP490 and EA9466) showed no statistically significant substrate effects (*p* > 0.05), indicating consistent performance across both materials. These substrate-dependent behaviors reflect complex interactions between surface chemistry, roughness, and adhesive wetting characteristics that require adhesive-specific optimization for multi-material assemblies.

## 3. Analytical Methods

Early shear-lag formulations assume that the adhesive carries load exclusively in shear and that adherends deform only in axial tension. Building on this, successive models introduce increasingly sophisticated treatments of bending effects, material nonlinearity, and adhesive thickness as follows:

- Volkersen shear-lag model (1938): the first analytical method to describe load transfer in single-lap joints, assuming linear elastic behavior of both adhesive and adherends and neglecting bending moments, leading to closed-form expressions for peak shear stress in the bond layer, dependent on the adherend, the adhesive elastic moduli, and on the joint geometry [36].
- Goland–Reissner plate-bending model (1944): extends Volkersen’s treatment by incorporating the bending moments induced by load eccentricity, yielding coupled expressions for shear and peel stresses through equilibrium of bent adherend plates connected by an elastic adhesive layer [40].
- Hart-Smith elastic–plastic model (1973): introduces adhesive plasticity by adopting an elastic-perfectly plastic stress–strain law for the adhesive and accounting for finite bending deformations of the adherends, producing a bending moment factor that reduces predicted edge stresses under high loads [41].
- Ojalvo & Eidinoff improved shear-strain model (1959): refines the shear-lag concept by employing a complete shear strain/displacement relation within the adhesive, demonstrating the influence of bondline thickness on stress distributions without substantial additional analytical complexity [42].

These foundational models serve as the basis for modern design and finite element analyses of bonded joints.

### 3.1. Volkersen Model

The Volkersen method, also known as the shear-lag model, is one of the best-known analytical models for stress analysis of bonded joints. It is the first analytical method of the simple shear-lag model for bonded joints known in the literature and was developed by Volkersen (1938) [43]. The model assumes perfectly linear elastic behavior of the adhesive layer and an interface continuity condition. The disadvantage of the Volkersen model in calculating the strength of a glued joint is that it does not account for bending moments. This means that the model considers that the adherends are deformed only in tension and the glued joint only in shear. The model equation can be written as follows:

(3) $$\tau =\frac{P\omega }{2b}\cdot \frac{\cosh\left(\omega x\right)}{sinh\left(\frac{\omega l}{2}\right)}+\left(\frac{t_{t}-t_{b}}{t_{t}+t_{b}}\right)\cdot \left(\frac{\omega l}{2}\right)\cdot \frac{\sinh\left(\omega x\right)}{cosh\left(\frac{\omega l}{2}\right)},$$

where parameter *ω* is given by the expression

(4) $$\omega =\sqrt{\frac{G_{a}}{Et_{t}t_{b}}}\left(1+\frac{t_{t}}{t_{b}}\right).$$

The origin of *x* is considered in the middle of the adhesive. *l* is the bonded area length, *b* is the bonded area width, *t_a_* is the thickness of the adhesive, *t_t_* is the top adherend thickness, *t_b_* is the bottom adherend thickness, *E* is the adherend tensile modulus, *G_a_* is the adhesive shear modulus, and *P* is the force applied at the end of the inner adherend.

### 3.2. Golland and Reissner Model

Golland and Reissner (1944) developed a more advanced analysis of the stress distribution in the bonded joint with consideration of the presence of bending moment (rotation of the adherends) [44]. The model uses the finite deflection theory of cylindrical bent plates to determine the forces at the edges of the bonded joint. The shear stress distribution in the adhesive τ is given as follows:

(5) $$\tau =-\frac{1}{8}\frac{\bar{P}}{c}\left\{\frac{\beta c}{t}\left(1+3k\right)\frac{\cosh\left(\frac{\beta c}{t}\frac{x}{c}\right)}{\sinh\left(\frac{\beta c}{t}\right)}+3\left(1-k\right)\right\},$$

where *t* is adherend thickness, *c* is half of the adherend overlap length, $\bar{P}$ is the applied tensile load (per unit width of the overlap), and *k* is the bending moment factor

(6) $$k=\frac{cosh(\alpha c)}{cosh\left(\alpha c\right)+2\sqrt{2}sinh(\alpha c)}\;,$$

where

(7) $$\alpha =\sqrt{\frac{3(1-\nu^{2})}{2}}\frac{1}{t}\sqrt{\frac{\bar{P}}{tE}}\;,\;\;\;\beta =\sqrt{8\frac{G_{a}t}{Et_{a}}}\;.$$

where ν is Poisson’s ratio of the adherend. The Golland and Reissner model also allows the determination of the distribution of peel stress by the equation

(8) $$\sigma =\frac{1}{∆}\frac{\bar{P}t}{c^{2}}\left[A+B\right],$$

where parameters *A* and *B* are given by equations

(9) $$A=\left(\lambda^{2}\frac{k}{2}(\cosh\left(\lambda \right)sin\left(\lambda \right)+\sinh\left(\lambda \right)cos\left(\lambda \right))+\lambda k^{\prime }\sinh\left(\lambda \right)sin\left(\lambda \right)\right)sinh\left(\frac{\lambda x}{c}\right)sin\left(\frac{\lambda x}{c}\right),$$

(10) $$B=\left(\lambda^{2}\frac{k}{2}(\sinh\left(\lambda \right)cos\left(\lambda \right)-\cosh\left(\lambda \right)sin\left(\lambda \right))+\lambda k^{\prime }\cosh\left(\lambda \right)cos\left(\lambda \right)\right)cosh\left(\frac{\lambda x}{c}\right)cos\left(\frac{\lambda x}{c}\right).$$

where *k’* is the factor of the transverse force, and factors *λ and Δ* are given by

(11) $$k^{\prime }=\frac{kc}{t}\sqrt{3(1-\nu^{2})\frac{\bar{P}}{tE}};\lambda =\frac{c}{t}\sqrt[{4}]{6\frac{E_{a}t}{Et_{a}}};\Delta =\frac{1}{2}\left(\sin\left(2\lambda \right)sinh(2\lambda )\right).$$

The selection of Volkersen and Goland–Reissner analytical models for this study is justified by several factors relevant to modern adhesive joint analysis. Despite their age, these models remain fundamental benchmarks in adhesive joint mechanics, providing closed-form solutions that enable rapid parametric analysis and design optimization without computational overhead. The Volkersen model establishes baseline stress predictions under pure shear assumptions, while the Goland–Reissner model incorporates bending effects crucial for understanding load eccentricity in single-lap configurations. These models serve as validation tools for finite element analysis and provide physical insight into stress distribution mechanisms. While acknowledging their limitations in capturing adhesive plasticity and complex material behavior, the analytical models enable direct comparison with the extensive literature data and provide design engineers with accessible tools for initial joint sizing. The systematic comparison between analytical predictions, nonlinear FEM results, and experimental data in this study quantifies the limitations of simplified analytical approaches for modern polymer adhesive systems, thereby informing practitioners about when more sophisticated analysis methods are required. This comparative approach enhances the practical utility of the research by providing multiple analysis perspectives relevant to different design phases and computational resource constraints.

## 4. Numerical Simulation

In the finite element study of single-lap bonded joints, two structural adhesives—3M Scotch-Weld™ DP620NS (polyurethane) and 3M Scotch-Weld™ DP490 (epoxy)—were selected based on their contrasting mechanical properties and processing characteristics. Loctite EA 9466 (epoxy) and Loctite HY 4090 (cyanoacrylate–epoxy hybrid) were excluded due to, respectively, inferior shear strength and impractical work life for specimen fabrication.

### 4.1. Determination of Mechanical Properties of the Selected Adhesives

**Figure 8:**
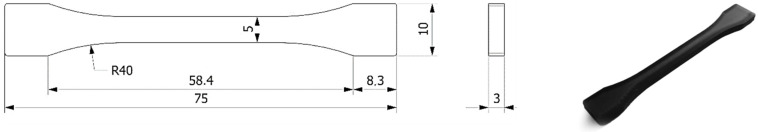
Geometry of adhesive tensile specimens—schematic of the specimen (**left**) and fabricated specimen (**right**).

The 3M DP620NS and 3M DP490 tensile specimens were fabricated in a mold made from PTFE sheets. Six samples were created for each type of adhesive. The geometry of the specimens was chosen according to EN ISO 527-2:2012 [45] as shown in Figure 8.

**Figure 9:**
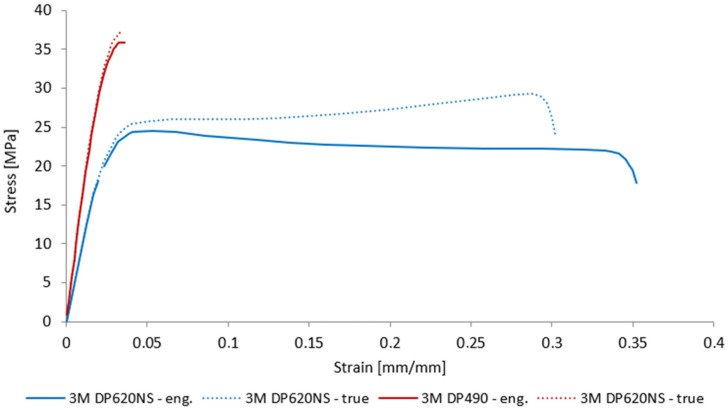
Measured true and engineering stress–strain tensile curves of the 3M DP490 and 3M DP620NS adhesives.

The tensile behavior of the selected adhesives was characterized using a Zwick-Roell Z250 electro-mechanical tensile testing machine equipped with 5 kN load cells and high-precision mechanical extensometers (designed specifically for soft materials and polymers). All tests were conducted at a constant crosshead speed of 2 mm/min, in accordance with ASTM D638-14 for plastics (Tensile Testing of Polymeric Materials) [46]. Figure 9 presents the mean engineering stress–strain curves obtained for each adhesive system. Both polyurethane (3M DP620NS) and epoxy (3M DP490) adhesives exhibit an initial linear increase in stress with strain, corresponding to elastic deformation. The elastic moduli, determined by the slope of this region between 0.1% and 0.5% strain, were found to be approximately 885 MPa for DP620NS and 1720 MPa for DP490, reflecting the higher stiffness of the epoxy adhesive. The peak (ultimate) tensile strengths were 23.8 MPa for DP620NS and 36 MPa for DP490.

### 4.2. FEM Modeling and Numerical Simulation of the Selected Adhesives

Finite element models were developed in Siemens Simcenter 3D (version 1847) to evaluate the interfacial shear $\tau_{xy}$ and peel $\sigma_{zz}$ (normal) stress distributions through the mid-thickness of the adhesive layer in single-lap joint specimens. Specimen geometries were meshed using second-order hexahedral elements (CHEXA20) with variable element sizes to balance accuracy and computational cost. A comprehensive mesh independence study was conducted to ensure the accuracy and reliability of the finite element results. The mesh density was systematically varied by refining the element size in critical regions, particularly at the overlap edges where stress concentrations occur. Four different mesh configurations were evaluated with the element size from 0.015 mm to 0.25 mm. The mesh convergence criterion was based on the maximum shear stress at the overlap edge, with convergence achieved when stress values varied by less than 2% between consecutive mesh refinements. The final mesh comprised approximately 95,000 elements with refined zones at the overlap edges (element size 0.05 mm), transitioning to coarser elements in regions of lower stress gradients (element size 0.3 mm). This approach ensured computational efficiency while maintaining accuracy in stress prediction, with the mesh-independent results showing excellent agreement with analytical predictions from the Volkersen model in the central overlap region.

Material properties for the adherends and adhesives were assigned according to experimentally validated elastic–plastic constitutive laws. Boundary conditions replicated the tensile test setup as follows: the lower clamp faces were fully constrained (*U_x_* = *U_y_* = *U_z_* = 0), the upper clamp faces were constrained in the loading direction (*U_z_* = 0), and the mean peak load from each specimen group was applied as a distributed load vector across the upper adherend surface.

**Figure 10:**
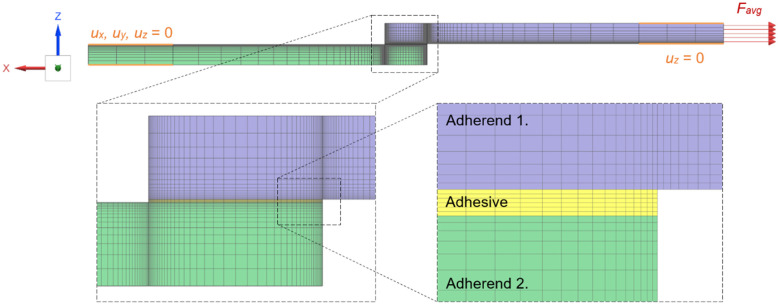
FEM model of single-shear-lap specimen with boundary conditions and detail of adherend overlap area.

Figure 10 illustrates the refined FEM mesh and boundary-condition application. These results underscore the critical role of mesh density within the adhesive layer and the necessity of accurately capturing edge effects when predicting joint strength and failure initiation in structural adhesive applications.

In order to assess and validate analytical predictions of adhesively bonded lap joints, both a purely linear (elastic) analysis and a fully nonlinear analysis (including material and geometric nonlinearities) were performed using the NX Nastran solver.

The linear study employed the SOL 101 “Linear Statics” solution sequence in NX Nastran, which assumes all materials exhibit small-strain, linear elastic behavior and neglects any geometric deformations beyond first order. Key aspects include constitutive law (Hooke’s law for adherends and adhesives, defined by Young’s modulus and Poisson’s ratio), static load application (a static load vector equal to the experimentally measured peak load was distributed across the upper adherend surface), and linear contact treatment (“Glued” bonded contact condition ensuring displacement continuity at the adhesive interfaces without allowing separation or slip). This approach yields stress distributions that can be directly compared to closed-form elastic joint models (e.g., shear-lag theory), serving both to validate mesh discretization and to quantify the deviation when nonlinear effects are introduced.

**Figure 11:**
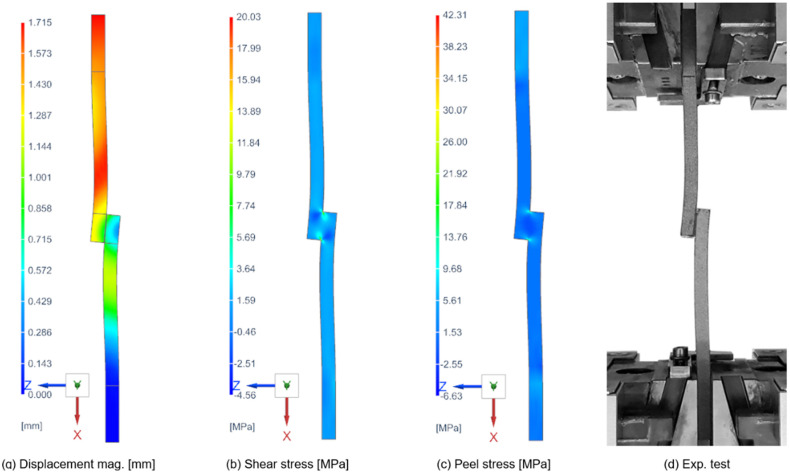
Numerical simulation of the single-lap-shear specimen (PA12 adherend + 3M DP490 adhesive).

To capture the true mechanical response of the joint (where the adhesive may yield and the joint geometry deforms under load), a multi-step nonlinear analysis was conducted using SOL 401 Multi-Step Nonlinear. This solver enables the following: material nonlinearity (plasticity models for adhesives, allowing the stress–strain curve to govern load transfer beyond yield), geometric nonlinearity (large-deformation (large-strain) formulations that account for stiffening or softening due to changing geometry under load), and subcase chaining (definition of sequential solution steps within a single run). Results of the numerical simulation of the single-lap-shear specimens are given in Figure 11.

## 5. Results and Discussion

**Figure 12:**
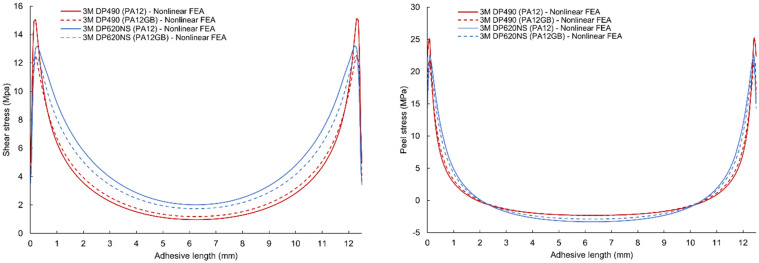
Shear and peel stress results of nonlinear finite element analysis for combinations of 3M DP490 and 3M DP620NS adhesives with PA12 and PA12GB adherends.

The simulations reveal that *τ_xy_* exhibits a symmetric distribution, peaking at the free edges of the overlap before decaying toward the joint center, whereas $\sigma_{zz}$ is antisymmetric, with tensile peel stresses at one edge and compressive peel stresses at the opposite edge—both effects characteristic of the free-edge phenomenon in adhesively bonded joints [47]. The shear and peel stress results along the centerline of the middle plane of the adhesive bond layer for nonlinear numerical simulation are given in Figure 12. Individual types of adhesives were computed for their critical forces obtained from experimental measurements in 50 incremental steps. The graphs show that maximum shear and peel stress along the centerline of the adhesive occurs at a very small distance from the free edge and then rapidly falls toward the center. The shear stress peak value of the DP490 epoxy adhesive with PA12 adherends is 17.4% (9.2% for PA12 GB adherends) higher than the DP620NS polyurethane adhesive, even though the critical force is 25.6% lower (18.7% for PA12 GB adherends). Peel stress results have very similar characteristics and ratios.

**Figure 13:**
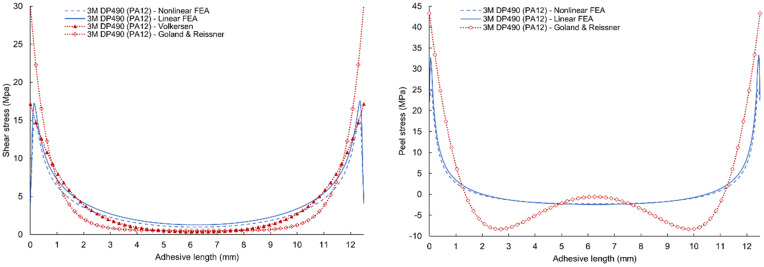
Comparison of shear and peel stress results of linear and nonlinear finite element analysis, Volkersen and Goland & Reissner analytical model for 3M DP490 adhesives with PA12 adherends.

**Figure 14:**
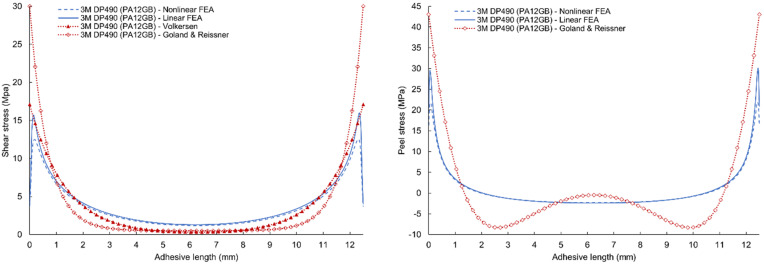
Comparison of shear and peel stress results of linear and nonlinear FEA, Volkersen, and Goland & Reissner analytical model for 3M DP490 adhesives with PA12GB adherends.

A comparison of the results of linear and nonlinear numerical simulation and analytical models according to Volkersen and Goland & Reissner for 3M DP490 epoxy adhesives are given in Figure 13 (for PA12 adherends) and Figure 14 (for PA12 GB adherends). Volkersen’s analytical model is the closest to the line of the linear FEA shear stress curve. The Goland & Reissner model is much closer to the results of linear FEA at the maximum values of peel stress. However, the results do not agree with the central area where the analytical model is characterized by a fall in stress. The shape of this part of the curve is affected by several parameters such as the thickness of the adherends, their flexural stiffness, and the flexibility of the adhesive. Discrepancies between analytical (Volkersen, Goland–Reissner) predictions and FEA arise principally because classical closed-form models neglect adhesive nonlinearity (plasticity) and the consequent load redistribution, as well as detailed adherend bending/stiffness effects. Modern experimental and numerical studies show that ductile adhesive yielding reduces edge peak shear stresses and smooths the stress gradient across the overlap; therefore, elastic analytical bounds tend to overestimate local peaks for tough adhesives. This necessitates nonlinear (elastic–plastic) FEA or experimentally calibrated models for accurate edge-stress and failure predictions in modern adhesive systems [48].

**Figure 15:**
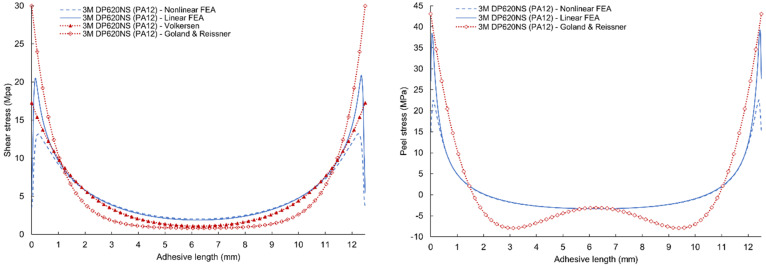
Comparison of shear and peel stress results of linear and nonlinear FEA, Volkersen and Goland & Reissner analytical model for 3M DP620NS adhesives with PA12 adherends.

**Figure 16:**
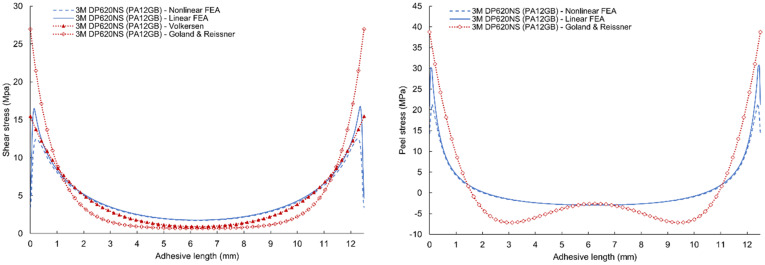
Comparison of shear and peel stress results of linear and nonlinear FEA, Volkersen, and Goland & Reissner analytical model for 3M DP620NS adhesives with PA12GB adherends.

Comparison of the numerical simulations and analytical models are given in Figure 15 and Figure 16 with very similar characteristics. However, a change is visible in the comparison of peel stresses, where a flattening of the curve can be seen due to the use of an adhesive with higher ductility.

The bondline thickness variation (0.19–0.23 mm) represents a 21% range that could potentially influence joint strength. Previous research indicates that adhesive joint strength is sensitive to bondline thickness, with optimal thickness typically ranging from 0.1 to 0.3 mm depending on adhesive type and joint geometry [49]. To assess this sensitivity, correlation analysis was performed between individual specimen bondline thickness and failure load. The results showed weak correlation (R^2^ < 0.15) for all adhesive types, suggesting that thickness variations within the observed range had minimal impact on joint strength. This finding is consistent with the relatively uniform stress distribution predicted by the FEM analysis in the central overlap region, where thickness variations have less influence compared to edge effects. The controlled application method using alignment jigs ensured adequate thickness uniformity, with the coefficient of variation remaining below 8% for all specimen groups. Future studies should investigate the performance limits at thickness extremes (<0.1 mm and >0.5 mm) to establish design guidelines for industrial applications.

The maximum measured shear strength of 5.0 MPa for DP620NS adhesive provides a useful benchmark for comparison with alternative joining methods for MJF-printed PA12 components. Mechanical fastening using self-tapping screws typically achieves pull-out strengths of 8–15 MPa in PA12 substrates, depending on screw geometry and installation torque, but introduces stress concentrations and requires precise hole positioning. Ultrasonic welding of PA12 components can achieve joint strengths of 15–25 MPa in properly designed joints with energy director features, but is limited to specific geometries and requires specialized equipment. Solvent-based bonding methods specific to polyamides can potentially achieve higher strengths (10–20 MPa) but involve hazardous chemicals and complex surface preparation procedures. The adhesive bonding approach offers advantages in terms of stress distribution, design flexibility, and processing simplicity, making it suitable for non-critical structural applications, rapid prototyping, and component assembly where moderate strength requirements (3–5 MPa) are acceptable. For load-bearing applications requiring higher strength, hybrid joining approaches combining adhesive bonding with mechanical fasteners should be considered to optimize both strength and reliability [50,51].

## 6. Conclusions

The goal of the paper was to determine the shear strength of the adhesive bonding of two types of adherend materials. The adherend materials tested were HP MJF PA12 and PA12 with 50% of glass beads created using an HP Multi-Jet Fusion 4200 series printer. The adhesives used were four epoxy-based structural adhesives (Loctite EA 9466 and 3M Scotch-Weld DP490), hybrid cyanoacrylate–epoxy (LOCTITE HY 4090), and polyurethane (3M Scotch-Weld DP620NS).

The results from the experimental measurements showed that all the adhesives reached a relatively high shear strength with only a one-step bonding process. This means that no chemical (such as primers, solvents, etc.), mechanical, or other special surface treatment methods (such as plasma) were used. The experimental tests were performed according to ASTM D3163 [18]. Three-dimensional optical surface roughness measurements were performed to determine any surface differences between the adherend materials with a mismatch not exceeding 4.5%.

The polyurethane-based adhesive (3M Scotch-Weld DP620NS) demonstrated superior performance with maximum shear strengths of 5.0 ± 0.35 MPa for PA12 and 4.4 ± 0.03 MPa for PA12GB, representing 30% and 17% higher strength, respectively, compared to epoxy-based alternatives. The hybrid cyanoacrylate–epoxy adhesive (Loctite HY4090) was the only system showing improved performance with glass-bead-reinforced substrate (16.5% increase from PA12 to PA12GB). Statistical analysis confirmed significant differences between adhesive types (F_3,24_ = 31.37, *p* < 0.001) with adhesive selection accounting for 65.7% of the total performance variance.

The practical implications of these findings extend across multiple industrial sectors where MJF-printed PA12 components are increasingly utilized. In the automotive industry, the demonstrated shear strengths of 3.6–5.0 MPa are sufficient for non-structural applications such as interior trim components, brackets, and electronic housings, where adhesive bonding offers weight reduction compared to mechanical fasteners. For aerospace applications, the consistent performance of polyurethane-based adhesives (DP620NS) makes them suitable for secondary structural components and interior assemblies, where the combination of moderate strength and excellent environmental resistance is crucial. The biomedical sector can benefit from these findings for device assembly and prosthetic components, where PA12’s established biocompatibility combines with reliable adhesive joining for applications requiring sterilization resistance. The superior performance of DP620NS adhesive with both PA12 variants suggests its potential for production applications where mixed material designs combining neat and glass-bead-reinforced components are required. These results provide engineering design data essential for implementing adhesive-bonded MJF assemblies in load-bearing applications while maintaining appropriate safety factors.

Linear and nonlinear finite element analysis and two analytical models (Volkersen and Goland & Reissner) were used to determine the shear and peel stress along the adhesive layer path. These models provide useful edge-stress estimates but fail to capture mid-bond stress distributions and adhesive plasticity, underscoring the need for numerical simulation in complex AM geometries. The discrepancies between FEA and analytical predictions can be attributed to several factors not captured in the simplified analytical models. The Volkersen model assumes elastic behavior and neglects bending effects, while the Goland–Reissner model considers bending but maintains elastic assumptions. The incorporation of adhesive plasticity in the nonlinear FEA, particularly for the more ductile polyurethane adhesive (DP620NS), results in stress redistribution and a reduction in peak stress concentrations at overlap edges. The adherend flexibility, especially significant in the relatively thin PA12 and PA12GB substrates, introduces geometric nonlinearity not accounted for in the analytical models. This flexibility leads to increased peel stress development and modified stress distributions compared to the rigid adherend assumptions in classical analyses. The FEA results show stress flattening in the central overlap region and reduced peak stresses at edges when material nonlinearity is considered, explaining the better correlation with experimental failure loads. These findings emphasize the importance of nonlinear analysis for accurate prediction of joint behavior in polymer-based adhesive systems, where both adhesive and adherend deformation contribute significantly to overall joint response.

## Figures and Tables

**Table 1:** Properties of the HP MJF PA12 and PA12 GB powders [21,23].

	Bulk Density [g/cm^3^]	Powder Melting Point [°C]	Particle Size [μm]
HP MJF 3D High Reusability PA12 powder	0.425	187	60
HP MJF 3D High Reusability PA12 GB powder	0.480	186	58

**Table 2:** Properties of the used plastic abrasive MF 30/40.

	Bulk Density [g/cm^3^]	Grain Size [mm]	Grain Shape [-]	Hardness [Mohs]	pH [-]	Fe Content [%]
PA MF 30/40	0.95	0.43–0.6	square	4.0	6–8	max 0.005

**Table 3:** Properties of surface of HP MJF PA12 and PA12 GB.

Name	Value [μm]		Description
	HP MJF PA12	HP MJF PA12 GB	
Ra	10.69	10.19	Average roughness of profile
Rq	14.02	13.52	Root mean square roughness of profile
Rz	103.39	97.61	Mean peak to valley height of roughness profile
Rt	130.81	132.36	Maximum peak to valley height of roughness profile
Rp	78.91	88.88	The maximum peak height of the roughness profile

**Table 4:** Key advantages of selected adhesives.

Adhesive	Chemistry	Key Advantages
Loctite EA 9466	Two-component epoxy	High-toughness, shear and peel strength, and chemical and electrical resistance
3M Scotch-Weld DP490	Modified epoxy	Thixotropic, high-toughness, low shrinkage, and wide substrate compatibility
3M Scotch-Weld DP620NS	Two-component polyurethane	Flexible, impact-resistant, good peel and shear strength, and low temp and low shrinkage
Loctite HY 4090	Cyanoacrylate–epoxy hybrid	Fast cure, high temp/moisture resistance, shock-resistant, and fills gaps

**Table 5:** Comparison of physical properties of selected adhesives (cured) [37].

Adhesive	LOCTITE EA 9466	3M Scotch-Weld DP490	LOCTITE HY 4090	3M Scotch-Weld DP620NS
Technology	Epoxy	Epoxy	Cyanoacrylate/Epoxy Hybrid	Polyurethane
Specific Gravity [g/cm^3^]	1.0	1.0	1.035	1.16
Tensile Modulus [MPa]	1718	1621	565	903
Tensile Strength (at break) [MPa]	32	35.9	7.1	24
Elongation (at break) [%]	3.0	2.97	3.6	36
Shore Hardness [Durometer D]	60	79	65–69	50
Glass Transition Temperature [°C]	62	69	88	60.2
Coefficient of Thermal Expansion [K^−1^]	-	64 × 10^−6^	71 × 10^−6^	-
Temperature Range [°C]	−55 to +120	−55 to +120	−40 to +150	−51 to +121

**Table 6:** Mean tensile shear strength and standard deviations for four structural adhesives on PA12 and PA12GB adherends. Values represent means of four replicates per cell (*n* = 4). Standard deviations indicate within-group measurement variability.

Adhesive	PA12 Mean ± SD [MPa]	PA12GB Mean ± SD [MPa]
3M Scotch-Weld DP620NS	5.003 ± 0.248	4.393 ± 0.019
3M Scotch-Weld DP490	3.712 ± 0.245	3.627 ± 0.124
Loctite EA 9466	3.655 ± 0.135	3.650 ± 0.301
Loctite HY 4090	3.849 ± 0.256	4.485 ± 0.133

**Table 7:** Two-way ANOVA results for tensile shear strength as a function of adhesive type and adherent material. Effect sizes (η^2^) calculated as SS_effect/SS_total. Critical F-values: F_3,24,0.05_ = 3.01, F_1,24,0.05_ = 4.26.

Source	SS	df	MS	F	*p*	Effect Size (η^2^)
Adhesive	5.888	3	1.963	31.37	<0.0001	0.657
Adherent	0.002	1	0.002	0.033	0.858	0.0002
Interaction	1.567	3	0.522	8.35	<0.001	0.175
Error	1.502	24	0.063	–	–	–
Total	8.958	31	–	–	–	–

## Data Availability

All raw and processed data supporting the findings of this study—including tensile test CSV files, 3D surface roughness maps, finite element input decks, and result files have been deposited in the Zenodo repository under the open license CC-BY 4.0 and can be accessed via the persistent identifier: 10.5281/zenodo.16310168. Additional data or code necessary to reproduce the analyses are available from the corresponding author upon reasonable request.
